# Antecedents, clinical and psychological characteristics of a large sample of individuals who have self-harmed recruited from primary care and hospital settings in Pakistan

**DOI:** 10.1192/bjo.2023.581

**Published:** 2023-11-13

**Authors:** Muhammad Omair Husain, Nasim Chaudhry, Tayyeba Kiran, Peter Taylor, Sehrish Tofique, Ayesha Khaliq, Amna Naureen, Suleman Shakoor, Paul Bassett, Shehla Naeem Zafar, Imran B. Chaudhry, Nusrat Husain

**Affiliations:** Campbell Family Mental Health Research Institute, Centre for Addiction and Mental Health, Toronto, Canada; Department of Psychiatry, Temerty Faculty of Medicine, University of Toronto, Canada; and Pakistan Institute of Living and Learning, Karachi, Pakistan; Pakistan Institute of Living and Learning, Karachi, Pakistan; Division of Psychology and Mental Health, School of Health Sciences, Faculty of Biology, Medicine and Health, University of Manchester, UK; Statsconsultancy Ltd, Amersham, UK; Iqra University Bharia, Karachi, Pakistan; University of Manchester, UK; and Department of Psychiatry, Ziauddin Hospital, Karachi, Pakistan

**Keywords:** Suicide, suicidal ideation, self-harm, Pakistan, depression

## Abstract

**Background:**

Suicide is one of the leading causes of mortality worldwide, and the majority of suicide deaths occur in low- and middle-income countries.

**Aims:**

To evaluate the demographic and clinical characteristics of individuals who have presented to health services following self-harm in Pakistan.

**Method:**

This study is a cross-sectional baseline analysis of participants from a large multicentre randomised controlled trial of self-harm prevention in Pakistan. A total of 901 participants with a history of self-harm were recruited from primary care clinics, emergency departments and general hospitals in five major cities in Pakistan. The Beck Scale for Suicide Ideation (BSI), Beck Depression Inventory (BDI), Beck Hopelessness Scale (BHS) and Suicide Attempt Self Injury Interview assessment scales were completed.

**Results:**

Most participants recruited were females (*n* = 544, 60.4%) in their 20s. Compared with males, females had lower educational attainment and higher unemployment rates and reported higher severity scores on BSI, BDI and BHS. Interpersonal conflict was the most frequently cited antecedent to self-harm, followed by financial difficulties in both community and hospital settings. Suicide was the most frequently reported motive of self-harm (*N* = 776, 86.1%). Suicidal intent was proportionally higher in community-presenting patients (community: *N* = 318, 96.9% *v.* hospital: *N* = 458, 79.9%; *P* < 0.001). The most frequently reported methods of self-harm were ingestion of pesticides and toxic chemicals.

**Conclusions:**

Young females are the dominant demographic group in this population and are more likely to attend community settings to seek help. Suicidal intent as the motivator of self-harm and use of potentially lethal methods may suggest that this population is at high risk of suicide.

Suicide is among the leading causes of mortality worldwide, claiming more than 800 000 lives per year.^1^ The majority of these deaths occur in low- and middle-income countries (LMIC), where there is a dearth of high-quality evidence on self-harm and suicide.^2^ Pooled data from five LMIC indicate a high prevalence of suicidal ideation in patients presenting to primary care facilities (one in ten patients), with approximately one in 45 people having attempted suicide.^3^ In Pakistan, a country where attempted suicide is still a criminal offence and religiously condemned, no official data on suicide and self-harm exist. From the limited literature available, there is evidence that suicide rates have been gradually rising in Pakistan.^4^

The United Nations Sustainable Development Goals include a reduction in mortality from non-communicable diseases, with suicide being one of the key indicators.^5^ Reduction in suicide is also part of the World Health Organization's mental health action plan.^6^ Suicide is a major public health issue across the world, with prevention strategies aiming to identify and provide intervention for those at highest risk. People with a history of self-harm are at significantly higher risk of suicide.^7,8^ In addition to suicide risk, self-harm is associated with substantial personal and societal cost.^9,10^ A robust evidence base is urgently needed to improve our understanding of self-harm and suicidal behaviours in LMIC settings. Such data have the potential to inform appropriate interventions and services to meet the needs of this group. Robust data to inform interventions are even more pertinent in low-income settings, where resources need to be used more judiciously.

The current evidence base on self-harm in Pakistan largely comprises small sample sizes and must be interpreted with caution, having limited generalisability to the wider population.^4^ A study of 221 individuals attending hospitals in Pakistan following self-harm found that such individuals often reported high suicidal intent (74% severe intent), were most likely to have used ingestion of pesticides as a means of self-harm (76%) and most often cited interpersonal difficulties (78–80%) as the key antecedent.^11^ The study also found that those who reported financial problems as the key antecedent were more likely to endorse greater depressive symptoms, suicidal ideation and hopelessness than those reporting interpersonal problems as the key antecedent. This study was, however, limited to a sample presenting to hospitals. The lack of data on self-harm and suicidal behaviours from primary care settings in Pakistan is particularly concerning, given that in low-resource settings patients are less likely to come into contact with secondary health services.

This study aimed to evaluate the demographic and clinical characteristics of a large sample of individuals who have presented to clinical services following self-harm in Pakistan, including both hospital and primary care services. In addition, we investigated differences in self-harm presentations between primary care and hospital settings, in terms of clinical and demographic characteristics. Finally, we examined differences between those who reported a previous history of self-harm in the past year and those who did not. In Western, higher-income countries, a history of multiple instances of self-harm has been associated with a higher repetition rate compared with cases without prior instances of self-harm.^12^ Young people with multiple self-harm instances report greater levels of psychological distress (greater depression and anxiety; lower self-esteem) than those who report a single episode of self-harm or no self-harm.^13^ These differences have not yet been widely studied in LMIC.

## Method

### Research design

This study is a cross-sectional baseline analysis of participants from a large multicentre randomised controlled trial of self-harm prevention in Pakistan (trial registration: NCT02742922). The trial focuses on the effectiveness of a culturally adapted manual assisted problem-solving intervention (C-MAP) following an episode of self-harm. The current study focuses on the baseline demographic, clinical and psychological characteristics of this group. Self-harm was defined as:

‘*an act with non-fatal outcome, in which an individual deliberately initiates a non-habitual behaviour that, without interventions from others, will cause self-harm, or deliberately ingests a substance in excess of the prescribed or generally recognised therapeutic dosage, and which is aimed at realizing changes which the subject desired via the actual or expected physical consequences*.’^14^

### Participants

A total of 901 patients, over the age of 18 years, living within the catchment area of the participating sites were recruited.

### Inclusion criteria

- All patients presented to the participating general practitioners or emergency departments or were admitted to the participating hospitals after an episode of self-harm.
- Patients aged 18 years and above were considered to be eligible for the study.
- Patients had to be living within the catchment area of the participating practices and hospitals.
- Patients did not need in-patient psychiatric treatment as determined by their clinical teams.

### Exclusion criteria

- Temporary residents unlikely to be available for follow-up.
- Patients with ICD-10: mental disorder secondary to a general medical condition or substance misuse; dementia; delirium; alcohol or drug dependence; schizophrenia; bipolar disorder; learning disability.
- Patients unable to engage with the invitation to participate or respond to the research questionnaires owing to a medical or psychiatric condition, or owing to living outside of the study catchment area.

### Setting

Ethics approval for the study was obtained from the Ethics Review Committee of Karachi Medical and Dental College (ref. 027/15) and the University of Manchester (ref: 2019-2610-10693). The authors assert that all procedures contributing to this work comply with the ethical standards of the relevant national and institutional committees on human experimentation and with the Helsinki Declaration of 1975, as revised in 2008. Patients were recruited from participating primary care clinics, emergency departments and general hospitals in five major urban centres from Pakistan: Karachi, Lahore, Peshawar, Quetta and Rawalpindi. The target population comprised all patients presenting to the participating sites following an episode of self-harm. Screening took place at participating general practices, emergency departments and medical wards of participating hospitals. After initially being introduced to the study by clinicians at the participating sites and obtaining verbal consent, researchers contacted potential participants. Potential participants were given detailed information about the research study, along with a participant information leaflet. If they met the inclusion criteria, they were invited to take part in the study. Written consent was obtained prior to enrolment in the study.

### Patient and public involvement and engagement (PPIE)

PPIE took place throughout the study period. PPIE informed the cultural adaptation and refinement of the experimental intervention that was examined in the primary study. PPIE also supported the recruitment and retention of participants, with trained patients involved in the development of consent forms and participant information sheets. Our patient group helped to devise recruitment and retention strategies such as the development of a locator form with multiple contact details for participants. The PPIE group helped to write lay summaries of the project for dissemination of findings and arranged community engagement events throughout the study period, with patients and their families participating in these events.

### Measures

All instruments were translated into Urdu and have previously been used in Pakistan.^11,15,16^ Assessments were completed in face-to-face sessions with participants. All questionnaires were administered by trained research staff. Research staff read questionnaires out for participants and marked participant responses on hard copies of the questionnaires.

Specific training was provided for each instrument by senior and experienced members of our research team. Monthly training included role play on how to administer the study instruments and interrater reliability sessions for each study instrument.

#### Demographic questionnaire

This study's specific form collected demographic information (age, sex, education, marital status, family status, employment status, monthly income and financial hardships).

#### Suicide Attempt Self-Injury Interview^17^

We collected information about the time (number of times self-harm has occurred in the past year and date of each episode), methods, antecedents, functions and circumstances of self-harm using an adapted version of the Suicide Attempt Self-Injury Interview. This semi-structured interview has been shown to have good interrater reliability (intraclass correlation coefficient = 0.96) and validity.^17^

#### Beck Scale for Suicide Ideation (BSI)^18^

The BSI is a self-report measure of current suicidal ideation. The 19-item instrument indicates the severity of suicidal ideation in the previous week. Higher scores (≥6) on the scale indicate greater risk.^19^ The convergent validity of the BSI has been demonstrated by measuring against other instruments of suicidal ideation (*r* = 0.41). We used the Urdu translated version of this instrument in prior work from Pakistan and found the Cronbach's alpha to be 0.89.^11^ The Cronbach's alpha for the BSI in the current study was 0.92.

#### Beck Hopelessness Scale (BHS)^20^

The BHS is a 20-item self-report instrument designed to measure three aspects of hopelessness: feelings about the future, loss of motivation and expectations. Higher scores indicate increasing severity of hopelessness: normal (0–3), mild hopelessness (4–8), moderate hopelessness (9–14) and severe hopelessness (>14). The test–retest scores of the scale have been found to be good (*r* = 0.81^21^). The Urdu version of the BHS has been used in Pakistan and reported to have a reliability coefficient (Kuder–Richardson index) of 0.93.^11^ The Cronbach's alpha for the BHS in the current study was 0.91.

#### Beck Depression Inventory (BDI)^22^

This is a 21-item scale of depressive symptoms. Higher scores on the scale indicate greater severity of depression. Mild depression is indicated by scores of 14 –19, moderate depression by scores of 20–28 and severe depression by scores of 29–63. The BDI has concurrent validity, correlating highly with the Hamilton Rating Scale for Depression (*r* = 0.72–0.73^23^). The test–retest reliability is also high (*r* = 0.60^24^). The Cronbach's alpha coefficients for the Urdu translated BDI are good (0.75–0.92^25^). We used the Urdu translated version of this instrument in our previous work; the Cronbach's alpha was 0.97.^11^ The Cronbach's alpha for the BDI in the current study was 0.90.

#### Statistical methods

Descriptive statistics were produced for all participants combined, and separately for those recruited from primary care and hospital settings. Continuous variables were summarised using the mean and standard deviation if normally distributed and using the median and interquartile range if not. Categorical variables were summarised using the number and percentage in each category. Statistical comparisons of the two settings (primary care and hospital) were made. Continuous variables were compared using unpaired *t*-tests if normally distributed and the Mann–Whitney test otherwise. The Mann–Whitney test was also used to compare ordinal measures between the two settings. Chi-squared test was used for categorical variables, except for variables with rare categories, where Fisher's exact test was preferred. Equivalent methods were used to compare the Beck scores between those with interpersonal and financial problems. Pearson correlation was used to examine the strengths of associations between the Beck component scores.

## Results

### Demographics

**Fig. 1:**
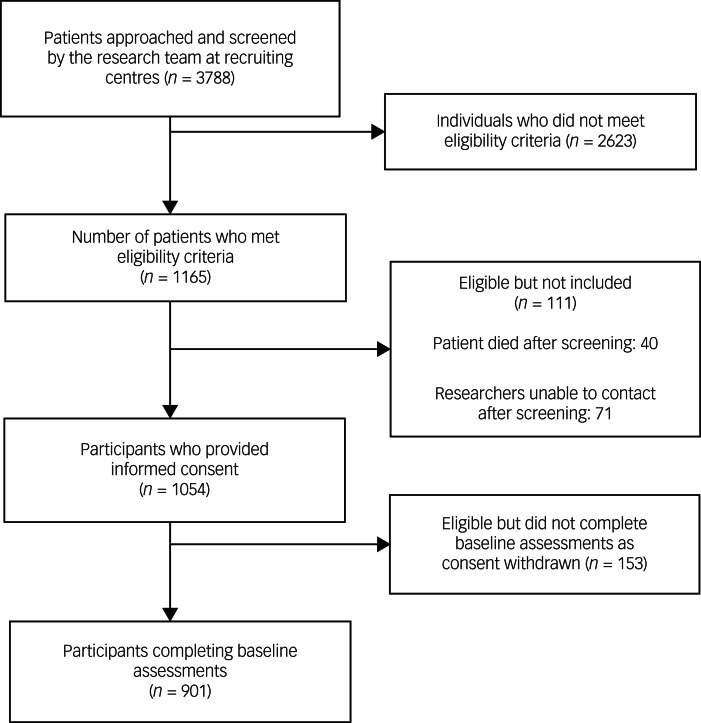
Participant flow diagram.

A total of 1165 patients met inclusion criteria for the primary study, of which 901 patients completed baseline measures between 12 March 2016 and 11 May 2018 (Fig. 1, participant flow diagram). Consent was withdrawn by 153 patients, 40 patients died after screening and 71 patients were not contactable following the initial screening. The demographic characteristics of the sample are outlined in Table 1. Most patients recruited from both primary care and hospitals and/or emergency departments were female (*N* = 544, 60.4%); however, proportionately fewer females presented to hospitals following self-harm (*N* = 302, 52.7%) compared with primary care settings (*N* = 242, 73.8%). The median age of the sample was 25 years; a significant proportion were between 18 and 25 years of age (*N* = 520, 57.7%). Most patients belonged to a nuclear family (*N* = 474, 52.6%), and the majority were married. The educational attainment of the sample was low, with 24.3% having no formal education (*N* = 219) and 67.8% having only completed up to 10 years of education (*N* = 611). Unemployment in the sample was high (*N* = 539; 59.8%), with almost half reporting being in debt (*N* = 444; 49.3%) and a significant proportion having difficulties in meeting day-to-day expenses (*N* = 566; 62.8%). Compared with males, females were less likely to be educated (no formal education: *N* = 169, 31.1%) and more likely to be unemployed (*N* = 438, 80.5%) and in debt (*N* = 284, 52.2%) and have difficulty in meeting day-to-day expenses (*N* = 383, 70.4%). Compared with males (*N* = 96, 26.9%), females (*N* = 250, 46.0%) were more likely to have slept hungry in the past month as a consequence of financial constraints (Supplementary Table 6 available at https://doi.org/10.1192/bjo.2023.581).

**Table 1:** Characteristics of the sample

	Total (*N* = 901)	Primary care setting (*n* = 328)	Hospital setting (*n* = 573)	*P*-value
	Median [IQR]			
Age (years)	25 [20, 30]	25 [21, 35]	23 [20, 30]	<0.001
Total monthly income (PKRs)	15 000 [10000, 30000]	10 000 [7650, 20000]	20 000 [12000, 40000]	<0.001
				*P-*value
Marital status	*N* (%)			
Single	305 (33.9%)	104 (31.7%)	201 (45.5%)	
Married	523 (58.1%)	196 (59.8%)	327 (57.1%)	0.58
Separated/divorced/widowed	73 (8.1%)	28 (8.5%)	45 (7.9%)	
Gender				
Male	357 (39.6%)	86 (26.2%)	271 (47.3%)	<0.001
Female	544 (60.4%)	242 (73.8%)	302 (52.7%)	
Family status				
Nuclear	474 (52.6%)	174 (53.0%)	300 (52.4%)	0.84
Joint	427 (47.4%)	154 (47.0%)	273 (47.6%)	
Education				
No formal education	219 (24.3%)	116 (36.3%)	105 (18.3%)	
Primary–secondary	392 (43.5%)	149 (45.4%)	243 (42.4%)	<0.001
Matric (lower secondary)–inter (upper secondary)	246 (27.3%)	51 (15.6%)	195 (34.0%)	
BA–Masters	44 (4.9%)	12 (3.7%)	32 (5.6%)	
Employment				
No	539 (59.8%)	224 (68.3%)	315 (55.0%)	<0.001
Yes	362 (40.2%)	104 (31.7%)	258 (45.0%)	
Antecedent of self-harm				
Interpersonal conflict	703 (78.0%)	249 (75.9%)	454 (79.2%)	
Financial difficulties	169 (18.8%)	72 (22.0%)	97 (16.9%)	
Illness	8 (0.9%)	0 (0.0%)	8 (1.4%)	
Bereavement	6 (0.7%)	3 (0.9%)	3 (0.5%)	0.11
Failure/fear of failure in exams	10 (1.1%)	3 (0.9%)	7 (1.2%)	
Trauma	2 (0.2%)	0 (0.0%)	2 (0.2%)	
Other	3 (0.3%)	1 (0.3%)	2 (0.3%)	
Do you have any debt?				
No	457 (50.7%)	131 (39.9%)	326 (56.9%)	<0.001
Yes	444 (49.3%)	197 (60.1%)	247 (43.1%)	
Difficulty in meeting day-to-day expenses in the past month				
No	335 (37.2%)	55 (16.8%)	280 (48.9%)	<0.001
Yes	566 (62.8%)	273 (83.2%)	293 (51.1%)	
Slept hungry in the past month				
No	555 (61.6%)	146 (44.5%)	409 (71.4%)	<0.001
Yes	346 (38.4%)	182 (55.5%)	164 (28.6%)	
Communicated self-harm				
No	594 (65.9%)	184 (56.1%)	410 (71.6%)	
Indirect communication	35 (3.9%)	6 (1.8%)	29 (5.1%)	<0.001
Direct communication	272 (30.2%)	138 (42.1%)	134 (23.4%)	
Intent to die				
No intent/minimal	125 (13.9%)	10 (3.1%)	115 (20.1%)	
Definite intent/ambivalence	220 (24.4%)	63 (19.2%)	157 (27.4%)	<0.001
Serious/extreme intent	556 (61.7%)	255 (77.7%)	301 (52.5%)	
Method of self-harm				
Pesticide	403 (44.7%)	93 (28.4%)	310 (54.1%)	
Ingestion of toxic chemicals	291 (32.3%)	175 (53.4%)	116 (20.2%)	
Ingestion of medication	155 (17.2%)	34 (10.4%)	121 (21.1%)	<0.001
Others (gunshot and jumping from heights)	46 (5.1%)	25 (7.6%)	21 (3.7%)	
Ingestion of medication plus pesticides	6 (0.7%)	1 (0.3%)	5 (0.9%)	
Physical condition following episode of self-harm				
No effect/very mild effect.	45 (5.0%)	13 (4.0%)	32 (5.5%)	
Mild effect	122 (13.7%)	32 (9.8%)	90 (15.9%)	
Moderate effect	522 (58.4%)	246 (75.4%)	276 (48.7%)	<0.001
Severe effect	131 (14.7%)	24 (7.4%)	107 (18.9%)	
Very severe effect	67 (7.5%)	11 (3.4%)	56 (9.9%)	
Extremely severe effect	06 (0.7%)	–	06 (1.1%)	

IQR, interquartile range.

### Antecedents of self-harm

The most prominent antecedent of self-harm observed in the hospital settings was interpersonal conflict (e.g. conflict with husband, parents, in-laws or peers; or breakdown of relationships), reported by 454 (79.2%) patients. Primary care presentations similarly reported interpersonal conflict as the primary antecedent of self-harm (*N* = 249; 75.9%). In both primary care and hospital presentations, financial difficulties were the second most common antecedent of self-harm (*N* = 169; 18.8%). There were no statistically significant differences in the endorsement of these antecedents between primary care and hospital presentations. Females were proportionately more likely than males to report interpersonal difficulties as precipitants of self-harm (*F* = 449, 82.5%; *M* = 254, 71.2%; Supplementary Table 6).

### Intent, methods and severity of self-harm episode

Most of the patients who self-harmed reported having done so with intent to die (*N* = 776, 86.1%). Suicidal intent was proportionally higher in patients presenting to primary care (primary care: *N* = 318, 96.9% *v.* hospital: *N* = 458, 79.9%; *P* < 0.001). Females reported proportionally higher rates of suicidal intent than males (*M* = 287, 80.4%; *F* = 489, 89.9%; Supplementary Table 6). Most of the patients (*N* = 594, 65.9%) did not communicate their thoughts or plans of self-harm in advance. Patients in primary care (*N* = 184, 56.1%) were less likely to communicate thoughts or plans of self-harm in advance compared with hospital-presenting patients (*N* = 410, 71.6%). The most frequently reported methods of self-harm were ingestion of pesticides and toxic chemicals. Patients in primary care settings (*N* = 268, 81.8%) were proportionally more likely to have ingested pesticides and toxic chemicals than those presenting to hospitals (*N* = 426, 74.3%). The use of pesticides was more common in males (*M* = 195, 54.6%; *F* = 208, 38.2%), whereas the use of toxic chemicals was more common in females (*M* = 76, 21.3%; *F* = 215, 39.5%) (Supplementary Table 6). Severity of self-harm for most participants in both groups was moderate. Individuals with severe, very severe and extreme forms of self-harm were more likely to present to hospitals (Table 1).

### Severity of symptoms

More than two-thirds of the sample reported moderate-to-severe depressive symptoms (*N* = 596, 66.2%; Table 2). Approximately half of patients (*N* = 458, 50.8%; Table 2) reported moderate-to-severe hopelessness, and over half (*N* = 522, 57.9%; Table 2) reported high levels of suicidal ideation. Patients from primary care settings had higher mean scores for depressive symptoms, hopelessness and suicidal ideation (Table 2). Females were more likely than males to report higher severity of depressive symptoms (BDI ≥ 28: *F* = 230, 42.3%; *M* = 103, 28.9%), hopelessness (BHS ≥ 15: *F* = 195, 35.9%; *M* = 72, 20.2%) and suicidal ideation (BSI ≥ 6: *F* = 342, 62.9%; *M* = 180, 50.4%) (Supplementary Table 7). Suicidal ideation was highly positively correlated with depression (*r* = 0.50, *P* < 0.001) and hopelessness (*r* = 0.52, *P* < 0.001) (Table 2). Individuals reporting financial problems as antecedents of self-harm had higher mean scores for hopelessness and depression (Table 3).

**Table 2:** Correlation and severity levels of scores

	Total	Primary care setting	Hospital setting	Cohen's *d*	*P*-value
Beck Depression Inventory					
Mean ± s.d.	25.4 ± 12.2	28.2 ± 11.4	23.7 ± 12.3	0.37	<0.001
Minimal (≤13)	162 (18.0%)	37 (11.3%)	125 (21.8%)		
Mild (14–19)	143 (15.9%)	41 (12.5%)	102 (17.8%)		<0.001
Moderate (20–28)	263 (29.2%)	98 (29.9%)	165 (28.8%)		
Severe ≥28	333 (37.0%)	152 (46.3%)	181 (31.6%)		
Beck Hopelessness Inventory					
Mean ± s.d.	9.5 ± 6.0	11.1 ± 5.9	8.6 ± 6.9	0.41	<0.001
Minimal (≤3)	262 (29.1%)	84 (25.6%)	178 (31.1%)		
Mild (4–8)	181 (20.1%)	44 (13.4%)	137 (23.9%)		<0.001
Moderate (9–14)	191 (21.2%)	64 (19.5%)	127 (22.2%)		
Severe (>15)	267 (29.6%)	136 (41.5%)	131 (22.9%)		
Beck Suicidal Ideation Scale					
Median [IQR]	9 [2, 17]	13 [5, 20]	7 [0, 15]	0.52	<0.001
Low	379 (42.1%)	101 (30.8%)	278 (48.5%)		<0.001
High	522 (57.9%)	227 (69.2%)	295 (51.5%)		
		Scale	Pearson correlation		*P*-value
Beck suicidal ideation scores		Beck depression scores	0.50		<0.001
		Beck hopelessness scores	0.52		<0.001

IQR, interquartile range.

**Table 3:** Comparison of severity scores between subjects with different antecedents

	Interpersonal problems (*n* = 697)	Financial problems (*n* = 167)	*P*-value
	Mean ± s.d. or median [IQR]		
Suicidal ideation (BSI)	9 [2, 17]	9 [2, 18]	0.69
Hopelessness (BHS)	9.3 ± 6.0	10.6 ± 6.0	0.01
Depression (BDI)	24.9 ± 12.1	27.3 ± 12.0	0.02

BDI, Beck Depression Inventory; BHS, Beck Hopelessness Scale; BSI, Beck Scale for Suicide Ideation; IQR, interquartile range.

### Repetition of self-harm in the past year

Most patients (*N* = 806, 89.5%) reported a single episode of self-harm in the past year (Supplementary Table 4). We conducted analysis on the demographic, clinical and psychological differences between patients with single attempts and those with multiple attempts of self-harm in the past year (Supplementary Table 5). The mean depression score in the multiple-attempts group was higher, at 27.9 (s.d. ± 13.4) compared with 25.1 (s.d. ± 12.0) in the single-attempts group. Hopelessness and suicidal ideation were also higher in the multiple-attempts group, although this difference was not statistically significant. Ingestion of pesticides and toxic chemicals was proportionately lower in the multiple-attempts group compared with those with single attempts (*N* = 56, 59.0% *v. N* = 638, 79.1%). Self-poisoning with medication was proportionately more common in the multiple-attempts group (*N* = 25, 26.3%) compared with the single-attempts group (*N* = 130, 16.1%).

## Discussion

To our knowledge, this is one of the largest cross-sectional studies exploring the demographic, clinical and psychological correlates of patients presenting to primary care and hospital settings following an episode of self-harm. Our findings illustrate that young females are the prominent demographic group in this population and are more likely to attend primary care than hospital settings to seek help. Females also had higher severity scores on depression, hopelessness and suicidal ideation scales than males. These women were likely to have low educational attainment, to be unemployed and to experience serious financial difficulties. Antecedents of self-harm for both males and females were primarily related to interpersonal conflict, replicating the results of Husain and colleagues,^11^ although this was more pronounced in females. More than 85% of self-harm attempts were with suicidal intent, and the majority of those who made such attempts did not communicate this to anyone prior to self-harm. The most common method of self-harm was self-poisoning, with the majority presenting after ingestion of pesticides or toxic chemicals. These characteristics are in line with those reported by Husain and colleagues.^11^ Those presenting to primary care services had higher severity scores for depression and suicidal ideation compared with individuals presenting to hospitals. Severity of self-harm in both groups was predominantly moderate, although, as expected, those with more severe incidents of self-harm presented to hospital settings. Almost 90% of patients had presented with a single episode of self-harm in the prior year.

The World Health Organization's 2014 report on suicide prevention describes vulnerability to suicide as a confluence of risk factors that include social isolation, interpersonal conflict, unemployment, financial strain, harmful use of alcohol, psychiatric illness and family history of suicide, along with other factors and wider system issues.^1^ The integrated motivational–volitional model of suicidal behaviour^26^ similarly specifies how a combination of environmental factors and life events initially set the scene for the development of suicidal ideation and behaviour. In the present study, the majority of individuals attending services following self-harm were likely to be unemployed and to have financial difficulties and an inability to meet day-to-day expenses. The association between poverty and suicidal behaviour in LMIC has been well documented.^27^ Financial hardship was also commonly reported in the sample described by Husain and colleagues.^11^ Poverty may be a factor that is more pertinent in a lower-middle-income country such as Pakistan. Interpersonal conflict was reported as the most common antecedent of self-harm in the current study, consistent with our previous work in Pakistan^11^ and reports from neighbouring India^28,29^ and the UK.^30,31^ Notably, although those citing financial hardship as a key antecedent reported higher rates of depression than those citing interpersonal difficulties, levels of suicidal ideation and hopelessness remained comparable. This is in contrast to Husain and colleagues, who found greater suicidal ideation and hopelessness in those citing financial hardship as the main antecedent.^11^ It is possible that the inclusion of primary care settings, which are associated with more severe difficulties, attenuated the association between this antecedent and severity of hopelessness and suicidal ideation. Interventions to address interpersonal conflicts and financial hardship may contribute to self-harm and suicide prevention.

Consistent with previous work in Pakistan,^11^ individuals presenting following self-harm in our study had relatively high severity scores for depressive symptoms, hopelessness and suicidal ideation. Research from India has reported that depression is the most common comorbid psychiatric condition in patients who self-harm.^29^ Hopelessness is associated with a 35% increased risk of self-harm in the following 12 months and a 2.5% increased risk of suicide in the same time period, as reported by a large prospective study of patients presenting to emergency departments in the UK.^32^ We found significant differences between individuals who presented with a single episode of self-harm in the past year compared with individuals with multiple self-harm episodes. Our findings are consistent with those from Western, high-income countries, where multiple self-harm is associated with greater severity of depression, among a host of other factors.^12,13^ In our sample, the proportion who reported multiple self-harm episodes was much lower than in Western populations (e.g. 64% of those attending hospital with self-harm in the UK reported past self-harm;^31^). A large cohort study investigating the risk of repeat self-harm and suicide in patients presenting to hospital with self-harm in rural Sri Lanka reported a low incidence of repeat self-harm and subsequent suicide death, compared with data from high-income countries.^33^ To inform suicide prevention strategies in LMIC, a better understanding of the low incidence of repeat self-harm is needed. These findings from the present study could reflect a greater unwillingness to share details of previous self-harm, given the stigma and legal status of self-harm in Pakistan.

A recent meta-analysis has identified that self-poisoning is the most common method of self-harm.^34^ Literature from India^35^ and Pakistan^11^ demonstrates that pesticides are the most common method of self-harm in this region. The findings from our current study are consistent with this, as the ingestion of chemicals and pesticides was the most common method of self-harm in both primary care and hospital-presenting individuals. These results are in contrast to those from Western, high-income countries, where overdose via prescribed medications is more common.^36^ The availability of highly toxic chemicals and pesticides in Pakistan is a potentially key factor in why this method may be common. These lethal substances are currently easily accessible in both urban and rural settings. Better understanding of methods of self-harm is important and can inform risk formulation in the psychosocial assessment of individuals presenting following self-harm. Data from a multicentre study of self-harm in young people from the UK found that self-injury (cutting) was a greater predictor of repeated self-harm and suicide than self-poisoning.^37^ However, cutting as a method of self-harm in Pakistan appears to be uncommon in presentations of self-harm to primary care and hospital settings, as demonstrated by the current study and our previous work.^11^ The propensity of individuals in Pakistan to use toxic chemicals and pesticides as a means of self-harm indicates that restriction of access to these substances may have a significant impact on self-harm and suicide prevention.^38^

To our knowledge, this is the first study to examine the demographic, clinical and psychological correlates of individuals presenting following self-harm to primary care and hospital settings in Pakistan. Females who were married and lived in a nuclear family were more likely to attend primary care services following self-harm. The majority of the primary care presentations were individuals from a lower socioeconomic stratum and had completed fewer years of education. Debt and financial constraints were also more prominent in individuals presenting to primary care. It is possible that the issues of travel and access to care for these individuals mean that primary care services are favoured over hospitals. The added challenges faced by this group in terms of debt and financial constraints may account for the higher severity scores for depressive symptoms and suicidality compared with people presenting to hospitals. However, interpersonal antecedents remained the most commonly cited. This finding is at odds with existing literature from LMIC, where suicidal ideation was identified as being more commonly reported in those attending hospital facilities compared with primary care samples.^3^ Our findings suggest that there is a need to introduce self-harm and suicide prevention strategies in primary care services to meet the needs of patients who could potentially be at higher risk of suicide than individuals presenting to hospitals.

One of the strengths of this study was the large sample size and recruitment of participants from multiple centres, which improved the generalisability of our findings. We were also able to recruit participants presenting to hospital and primary care settings and used standardised clinical assessment instruments. However, our sample did not account for the cases of self-harm that were not presenting to healthcare services, nor did we have a control group with no history of self-harm to serve as a comparison. The present study recruited survivors of self-harm and there may potentially be underrepresentation of lethal forms that resulted in suicide. Although we drew comparisons between single episodes of self-harm and multiple episodes, the data collected were limited to episodes in the prior year and not reflective of lifetime history. We were, therefore, unable to ascertain what proportion of single episodes of self-harm were first episodes. Finally, as exploration of antecedents was retrospective in nature, there was potential for recall bias.

Suicide and self-harm are major public health concerns worldwide. Self-harm presentations in primary care and hospital settings allow the opportunity for intervention. Accurately identifying, appropriately assessing and providing evidence-based treatment to manage self-harm are key public health priorities. Meta-analysis has so far failed to demonstrate evidence for the utility of assessment scales in patients presenting with self-harm, however, recommending detailed psychosocial assessment as the cornerstone of treatment.^39^ The demographic, clinical and psychological characteristics we have identified are crucial in informing such assessments and hence appropriate interventions. The demographic, psychological and clinical correlates of self-harm in Pakistan are in some ways unique and differ from those in other contexts. Suicidal intent as the motivator of self-harm and use of potentially lethal methods may suggest that this population is at high risk of suicide. This may well also explain the relatively low rates of repetition of self-harm in the current sample; however, further research is necessary to definitively establish why self-harm repetition is uncommon in Pakistan and other LMIC. Detailed assessment and understanding of antecedents of self-harm are essential to identify appropriate suicide prevention interventions. Communication of self-harm was exceptionally low in the current study, consistent with our previous work in Pakistan. This is possibly related to the highly stigmatised nature of self-harm in Pakistan and has implications for prevention strategies, where screening tools may be needed to identify at-risk groups.

## Supporting information

Husain et al. supplementary material Husain et al. supplementary material

## Data Availability

Data are available upon reasonable request.

## References

[1] World Health Organization. Preventing Suicide: A Global Imperative. WHO, 2014.

[2] Varnik P. Suicide in the world. Int J Environ Res Public Health 2012; 9(3): 760–71. 22690161 10.3390/ijerph9030760 PMC3367275

[3] Jordans M, Rathod S, Fekadu A, Medhin G, Kigozi F, Kohrt B, et al. Suicidal ideation and behaviour among community and health care seeking populations in five low- and middle-income countries: a cross-sectional study. Epidemiol Psychiatr Sci 2018; 27(4): 393–402. 28202089 10.1017/S2045796017000038 PMC5559346

[4] Shekhani SS, Perveen S, Hashmi DE, Akbar K, Bachani S, Khan MM. Suicide and deliberate self-harm in Pakistan: a scoping review. BMC Psychiatry 2018; 18(1): 44. 29433468 10.1186/s12888-017-1586-6 PMC5809969

[5] United Nations. *Transforming Our World: The 2030 Agenda for Sustainable Development*. UN, 2015.

[6] World Health Organization. World Health Action Plan 2013–2020. WHO, 2013.

[7] Hawton K, Bergen H, Cooper J, Turnbull P, Waters K, Ness J, et al. Suicide following self-harm: findings from the Multicentre Study of Self-harm in England, 2000–2012. J Affect Disord 2015; 175: 147–51. 25617686 10.1016/j.jad.2014.12.062

[8] Ribeiro JD, Franklin JC, Fox KR, Bentley KH, Kleiman EM, Chang BP, et al. Self-injurious thoughts and behaviors as risk factors for future suicide ideation, attempts, and death: a meta-analysis of longitudinal studies. Psychol Med 2016; 46(2): 225–36. 26370729 10.1017/S0033291715001804 PMC4774896

[9] Tsiachristas A, McDaid D, Casey D, Brand F, Leal J, Park AL, et al. General hospital costs in England of medical and psychiatric care for patients who self-harm: a retrospective analysis. Lancet Psychiatry 2017; 4(10): 759–67. 28890321 10.1016/S2215-0366(17)30367-X PMC5614771

[10] Goldman-Mellor SJ, Caspi A, Harrington H, Hogan S, Nada-Raja S, Poulton R, et al. Suicide attempt in young people: a signal for long-term health care and social needs. JAMA Psychiatry 2014; 71(2): 119–27. 24306041 10.1001/jamapsychiatry.2013.2803 PMC3946312

[11] Husain MO, Umer M, Taylor P, Chaudhry N, Kiran T, Ansari S, et al. Demographic and psychosocial characteristics of self-harm: the Pakistan perspective. Psychiatry Res 2019; 279: 201–6. 30851986 10.1016/j.psychres.2019.02.070

[12] Chai Y, Luo H, Yip PSF. Prevalence and risk factors for repetition of non-fatal self-harm in Hong Kong, 2002–2016: a population-based cohort study. Lancet Reg Health West Pac 2020; 2: 100027. 34327378 10.1016/j.lanwpc.2020.100027 PMC8315465

[13] Madge N, Hawton K, McMahon EM, Corcoran P, De Leo D, de Wilde EJ, et al. Psychological characteristics, stressful life events and deliberate self-harm: findings from the Child & Adolescent Self-harm in Europe (CASE) Study. Eur Child Adolesc Psychiatry 2011; 20(10): 499–508. 21847620 10.1007/s00787-011-0210-4

[14] Schmidtke A, Bille-Brahe U, DeLeo D, Kerkhof A, Bjerke T, Crepet P, et al. Attempted suicide in Europe: rates, trends and sociodemographic characteristics of suicide attempters during the period 1989–1992. Results of the WHO/EURO multicentre study on parasuicide. Acta Psychiatr Scand 1996; 93(5): 327–38. 8792901 10.1111/j.1600-0447.1996.tb10656.x

[15] Husain N, Afsar S, Ara J, Fayyaz H, Rahman RU, Tomenson B, et al. Brief psychological intervention after self-harm: randomised controlled trial from Pakistan. Br J Psychiatry 2014; 204(6): 462–70. 24676964 10.1192/bjp.bp.113.138370

[16] Husain N, Chaudhry N, Fatima B, Husain M, Amin R, Chaudhry IB, et al. Antidepressant and group psychosocial treatment for depression: a rater blind exploratory RCT from a low income country. Behav Cogn Psychother 2014; 42(6): 693–705. 23867053 10.1017/S1352465813000441

[17] Linehan MM, Comtois KA, Brown MZ, Heard HL, Wagner A. Suicide Attempt Self-Injury Interview (SASII): development, reliability, and validity of a scale to assess suicide attempts and intentional self-injury. Psychol Assess 2006; 18(3): 303–12. 16953733 10.1037/1040-3590.18.3.303

[18] Beck AT, Steer RA. *BSI, Beck Scale for Suicide Ideation: Manual*. Psychological Corporation, 1991.

[19] de Beurs DP, Fokkema M, O'Connor RC. Optimizing the assessment of suicidal behavior: the application of curtailment techniques. J Affect Disord 2016; 196: 218–24. 26938964 10.1016/j.jad.2016.02.033

[20] Beck AT, Steer RA. BHS, Beck Hopelessness Scale: Manual. Psychological Corporation, 1988.

[21] Ayub N. Measuring hopelessness and life orientation in Pakistani adolescents. Crisis 2009; 30(3): 153–60. 19767271 10.1027/0227-5910.30.3.153

[22] Beck AT, Ward CH, Mendelson M, Mock J, Erbaugh J. An inventory for measuring depression. Arch Gen Psychiatry 1961; 4: 561–71. 13688369 10.1001/archpsyc.1961.01710120031004

[23] Beck AT, Steer RA, Carbin MG. Psychometric properties of the Beck Depression Inventory: twenty-five years of evaluation. Clin Psychol Rev 1988; 8: 77–100.

[24] Jo SA, Park MH, Jo I, Ryu SH, Han C. Usefulness of Beck Depression Inventory (BDI) in the Korean elderly population. Int J Geriatr Psychiatry 2007; 22(3): 218–23. 17044132 10.1002/gps.1664

[25] Khan AA, Marwat SK, Noor MM, Fatima S. Reliability and validity of Beck Depression Inventory among general population in Khyber Pakhtunkhwa, Pakistan. J Ayub Med Coll Abbottabad 2015; 27(3): 573–5. 26721010

[26] O'Connor RC, Kirtley OJ. The integrated motivational-volitional model of suicidal behaviour. Philos Trans R Soc Lond B Biol Sci 2018; 373(1754): 20170268. 10.1098/rstb.2017.0268 PMC6053985 30012735

[27] Iemmi V, Bantjes J, Coast E, Channer K, Leone T, McDaid D, et al. Suicide and poverty in low-income and middle-income countries: a systematic review. Lancet Psychiatry 2016; 3(8): 774–83. 27475770 10.1016/S2215-0366(16)30066-9

[28] Kattimani S, Sarkar S, Rajkumar RP, Menon V. Stressful life events, hopelessness, and coping strategies among impulsive suicide attempters. J Neurosci Rural Pract 2015; 6(2): 171–6. 25883475 10.4103/0976-3147.153222 PMC4387806

[29] Radhakrishnan R, Andrade C. Suicide: an Indian perspective. Indian J Psychiatry 2012; 54(4): 304–19. 23372232 10.4103/0019-5545.104793 PMC3554961

[30] Cooper J, Husain N, Webb R, Waheed W, Kapur N, Guthrie E, et al. Self-harm in the UK: differences between South Asians and Whites in rates, characteristics, provision of service and repetition. Soc Psychiatry Psychiatr Epidemiol 2006; 41(10): 782–8. 16838089 10.1007/s00127-006-0099-2

[31] Bickley H, Steeg S, Turnbull P, Haigh M, Donaldson I, Matthews V, et al. Self-Harm in Manchester January 2010 to December 2011. University of Manchester, 2013.

[32] Steeg S, Haigh M, Webb RT, Kapur N, Awenat Y, Gooding P, et al. The exacerbating influence of hopelessness on other known risk factors for repeat self-harm and suicide. J Affect Disord 2016; 190: 522–8. 26561943 10.1016/j.jad.2015.09.050

[33] Knipe D, Metcalfe C, Hawton K, Pearson M, Dawson A, Jayamanne S, et al. Risk of suicide and repeat self-harm after hospital attendance for non-fatal self-harm in Sri Lanka: a cohort study. Lancet Psychiatry 2019; 6(8): 659–66. 31272912 10.1016/S2215-0366(19)30214-7 PMC6639451

[34] Carroll R, Metcalfe C, Gunnell D. Hospital presenting self-harm and risk of fatal and non-fatal repetition: systematic review and meta-analysis. PLoS One 2014; 9(2): e89944. 24587141 10.1371/journal.pone.0089944 PMC3938547

[35] Grover S, Sarkar S, Bhalla A, Chakrabarti S, Avasthi A. Demographic, clinical and psychological characteristics of patients with self-harm behaviours attending an emergency department of a tertiary care hospital. Asian J Psychiatr 2016; 20: 3–10. 27025463 10.1016/j.ajp.2016.01.006

[36] Geulayov G, Kapur N, Turnbull P, Clements C, Waters K, Ness J, et al. Epidemiology and trends in non-fatal self-harm in three centres in England, 2000–2012: findings from the Multicentre Study of Self-harm in England. BMJ Open 2016; 6(4): e010538. 10.1136/bmjopen-2015-010538 PMC4854013 27130163

[37] Hawton K, Bergen H, Kapur N, Cooper J, Steeg S, Ness J, et al. Repetition of self-harm and suicide following self-harm in children and adolescents: findings from the Multicentre Study of Self-harm in England. J Child Psychol Psychiatry 2012; 53(12): 1212–9. 22537181 10.1111/j.1469-7610.2012.02559.x

[38] Zalsman G, Hawton K, Wasserman D, van Heeringen K, Arensman E, Sarchiapone M, et al. Suicide prevention strategies revisited: 10-year systematic review. Lancet Psychiatry 2016; 3(7): 646–59. 27289303 10.1016/S2215-0366(16)30030-X

[39] Chan MK, Bhatti H, Meader N, Stockton S, Evans J, O'Connor RC, et al. Predicting suicide following self-harm: systematic review of risk factors and risk scales. Br J Psychiatry 2016; 209(4): 277–83. 27340111 10.1192/bjp.bp.115.170050

