# Supplementary material for: Antecedents, clinical and psychological characteristics of a large sample of individuals who have self-harmed recruited from primary care and hospital settings in Pakistan
Source: BJPsych Open. 2023 Nov 13;9(6):e216. doi: 10.1192/bjo.2023.581 (PMC10753970; doi:10.1192/bjo.2023.581)
Supplement: Husain et al. supplementary material [file S2056472423005811sup001.docx]

**Supplementary Tables**

| **Table 4 Characteristics of the sample by number of self-harm episodes in the past year** | | | | | |
| --- | --- | --- | --- | --- | --- |
|  | **Total** | **One attempt** | | **2 or more attempt** |  |
|  | **(N – 901)** | **(N – 806)** | | **(N – 95)** | ***P-value*** |
|  | **Mean (SD) or Median [IQR]** | | |  |  |
| Age (years) | 25 [20, 30] | | 25 [20, 30] | 25 [20, 32] | .47 |
| Total monthly income (PKRs) | 15000 [10000, 30000] | | 15000 [10000, 30000] | 20000 [10000, 40000] | .11 |
| **Age (years)** |  | |  |  |  |
| 18 – 25 | 520(57.7%) | | 472(58.6%) | 48(50.5%) |  |
| 26 – 35 | 268(29.7%) | | 236(29.30%) | 32(33.7%) |  |
| 36 – 45 | 87(9.7%) | | 75(9.3%) | 12(12.6%) | .62 |
| 46 – 55 | 25(2.8%) | | 22(2.7%) | 3(2.2%) |  |
| 56 or above | 1(0.1%) | | 1(0.1%) | 0 (0.0%) |  |
|  |  | |  |  |  |
| **N (%)** | | | | | ***P-value*** |
|  |  | |  |  |  |
| **Marital status** |  | |  |  |  |
| Single | 305(33.9%) | | 279(34.6%) | 26(27.4%) |  |
| Married | 523(58.1%) | | 468(58.1%) | 55(57.9%) | .204 |
| Separated/Divorced/widow | 73(8.1%) | | 59(7.3%) | 14(14.7%) |  |
| **Gender** |  | |  |  |  |
| Male | 357(39.6%) | | 319(39.6%) | 38(40.0%) | 1.000 |
| Female | 544(60.4%) | | 487(60.4%) | 57(60.0%) |  |
| **Family status** |  | |  |  |  |
| Nuclear | 474(52.6%) | | 428(53.1%) | 46(48.4%) | .45 |
| Joint | 427(47.4%) | | 378(46.9%) | 49(51.6%) |  |
| **Education** |  | |  |  |  |
| No formal education | 219(24.3%) | | 202(25.1%) | 17(17.9%) |  |
| Primary - Secondary | 392(43.5%) | | 351(43.6%) | 41(43.2%) | .34 |
| Matric – Inter | 246(27.3%) | | 215(26.7%) | 31(32.6%) |  |
| BA - Masters | 44(4.9%) | | 38(4.7%) | 6(6.3%) |  |
| **Employment** |  | |  |  |  |
| No | 539(59.8%) | | 485(60.2%) | 54(56.8%) | .53 |
| Yes | 362(40.2%) | | 321(39.8%) | 41(43.2%) |  |
| **Antecedent of self-harm** |  | |  |  |  |
| Interpersonal conflict | 703(78.0%) | | 627(77.8%) | 76(80.0%) |  |
| Financial difficulties | 169(18.8%) | | 153(19.0%) | 16(16.8%) | .88 |
| Other (illness/death/failure in exam) | 29(3.2%) | | 26(3.2%) | 3(3.2%) |  |
| **Do you have any debt?** |  | |  |  |  |
| No | 457(50.7%) | | 404(50.1%) | 53(55.8%) | .329 |
| Yes | 444(49.3%) | | 402(49.9%) | 42(44.2%) |  |
| **Difficulty in meeting day to day expenses in the last month** |  | |  |  |  |
| No | 335(37.2%) | | 302(37.5%) | 33(34.7%) | .60 |
| Yes | 566(62.8%) | | 504(62.5%) | 62(65.3%) |  |
| **Slept hungry in the last month** |  | |  |  |  |
| No | 555(61.6%) | | 496(61.5%) | 59(62.1%) | 1.00 |
| Yes | 346(38.4%) | | 310(38.5%) | 36(37.9%) |  |
| **Communicated Self harm** |  | |  |  |  |
| No | 594(65.9%) | | 538(66.7%) | 56(58.9%) |  |
| Indirect communication | 35(3.9%) | | 30(3.7%) | 5(5.3%) | .30 |
| Direct communication | 272(30.2%) | | 238(29.5%) | 34(35.8%) |  |
| **Intent to die** |  | |  |  |  |
| No intent/minimal | 125(13.9%) | | 116(14.4%) | 9(9.5%) |  |
| Definite intent/ambivalence | 220(24.4%) | | 192(23.8%) | 28(29.5%) | .27 |
| Serious/extreme intent | 556(61.7%) | | 498(61.8%) | 58(61.1%) |  |
| **Method of self-harm** |  | |  |  |  |
| Pesticide | 403(44.7%) | | 371(46.0%) | 32(33.7%) |  |
| Ingestion of toxic chemicals | 291(32.3%) | | 267(33.1%) | 24(25.3%) |  |
| Ingestion of medication | 155(17.2%) | | 130(16.1%) | 25(26.3%) | <.001 |
| Others (gunshot and jumping from heights) | 46(5.1%) | | 35(4.3%) | 11(11.6%) |  |
| Ingestion of medication plus pesticides | 6(0.7%) | | 3(0.4%) | 3(3.2%) |  |

**Table 5**

| **Clinical outcome measures scores by number of self-harm episodes in the past year** | | | | |
| --- | --- | --- | --- | --- |
| **Beck Depression Inventory** | **Total** | **One attempt** | **Two or more attempts** | **P-value** |
| Mean ± SD | 25.4 ± 12.2 | 25.1 ± 12.0 | 27.9 ± 13.4 | 0.03 |
| Minimal ≤ 13 | 162(18.0%) | 147(18.2%) | 15(15.8%) |  |
| Mild 14 to 19 | 143(15.9%) | 132(16.4%) | 11(11.6%) | .04 |
| Moderate 20 to 28 | 263(29.2%) | 240(29.8%) | 23(24.2%) |  |
| Severe ≥ 28 | 333(37.0%) | 287(35.6%) | 46(48.4%) |  |
| **Beck Hopelessness Inventory** |  |  |  |  |
| Mean ± SD | 9.5 ± 6.0 | 9.4 ± 6.0 | 10.2 ± 5.6 | 0.23 |
| Minimal <=3 | 262(29.5%) | 238(29.5%) | 24(25.3%) |  |
| Mild 4 to 8 | 181(20.1%) | 168(20.8%) | 13(13.7%) | .36 |
| Mod 9 to 14 | 191(21.2%) | 159(19.7%) | 32(33.7%) |  |
| Severe >15 | 267(29.6%) | 241(29.9%) | 26(27.4%) |  |
| **Beck Suicidal Ideation Scale** |  |  |  |  |
| Median [IQR] | 9 [2, 17] | 9 [2, 17] | 10 [0, 19] | 0.92 |
| Low | 379(42.1%) | 337(41.8%) | 42(44.2%) | .65 |
| High | 522(57.9%) | 469(58.2%) | 53(55.8%) |  |

| **Table 6**  **Characteristics of the sample by gender** | | | | | |
| --- | --- | --- | --- | --- | --- |
|  | **Total** | **Male** | | **Female** |  |
|  | **(N – 901)** | **(n – 359)** | | **(n – 542)** | ***P-value*** |
|  | **Mean ± SD or Median [IQR]** | | |  |  |
| Age (years) | 25 [20, 30] | | 22 [20, 29] | 25 [21, 32] | <.001 |
| Total monthly income (PKRs) | 15000 [10000, 30000] | | 22000 [15000, 40000] | 12000 [8000, 25000] | <.001 |
| **Age (years)** |  | |  |  |  |
| 18 – 25 | 520(57.7%) | | 232(65.0%) | 288(52.9%) |  |
| 26 – 35 | 268(29.7%) | | 82(23.0%) | 186(34.2%) |  |
| 36 – 45 | 87(9.7%) | | 29(8.1%) | 58(10.7%) | .001 |
| 46 – 55 | 25(2.8%) | | 13(3.6%) | 12(2.2%) |  |
| 56 or above | 1(0.1%) | | 1(0.3%) | 0(0.0%) |  |
|  |  | |  |  |  |
| **n (%)** | | | | | ***P-value*** |
|  |  | |  |  |  |
| **Marital status** |  | |  |  |  |
| Single | 305(33.9%) | | 184(51.5%) | 121(22.4%) |  |
| Married | 523(58.1%) | | 145(40.6%) | 378(69.5%) | <.001 |
| Separated/Divorced/widow | 73(8.1%) | | 28(7.8%) | 45(8.3%) |  |
| **Family status** |  | |  |  |  |
| Nuclear | 474(52.6%) | | 199(55.7%) | 275(50.6%) | .13 |
| Joint | 427(47.4%) | | 158(44.3%) | 269(49.4%) |  |
| **Education** |  | |  |  |  |
| No formal education | 219(24.3%) | | 50(14.0%) | 169(31.1%) |  |
| Primary - Secondary | 392(43.5%) | | 171(47.9%) | 221(40.6%) | <.001 |
| Matric – Inter | 246(27.3%) | | 120(33.6%) | 126(23.2%) |  |
| BA - Masters | 44(4.9%) | | 16(4.5%) | 28(5.2%) |  |
| **Community or emergency department** |  | |  |  |  |
| Emergency | 573(63.6%) | | 271(75.9%) | 302(55.5%) | <.001 |
| Community | 328(36.4%) | | 86(24.1%) | 242(44.5%) |  |
| **Employment** |  | |  |  |  |
| No | 539(59.8%) | | 101(28.3%) | 438(80.5%) | <.001 |
| Yes | 362(40.2%) | | 256(71.7%) | 106(19.5%) |  |
| **Antecedent of self-harm** |  | |  |  |  |
| Interpersonal conflict | 703(78.0%) | | 254(71.2%) | 449(82.5%) |  |
| Financial difficulties | 169(18.8%) | | 85(23.8%) | 84(15.4%) | <.001 |
| Other (illness/death/failure in exam) | 29(3.2%) | | 18(5.0%) | 11(2.0%) |  |
|  |  | |  |  |  |
| **Do you have any debt?** |  | |  |  |  |
| No | 457(50.7%) | | 197(55.2%) | 260(47.8%) | .03 |
| Yes | 444(49.3%) | | 160(44.8%) | 284(52.2%) |  |
| **Difficulty in meeting day to day expenses in the last month** |  | |  |  |  |
| No | 335(37.2%) | | 174(48.7%) | 161(29.6%) | <.001 |
| Yes | 566(62.8%) | | 183(51.3%) | 383(70.4%) |  |
| **Slept hungry in the last month** |  | |  |  |  |
| No | 555(61.6%) | | 261(73.1%) | 294(54.0%) | <.001 |
| Yes | 346(38.4%) | | 96(26.9%) | 250(46.0%) |  |
| **Communicated Self harm** |  | |  |  |  |
| No | 594(65.9%) | | 247(69.2%) | 347(63.8%) |  |
| Indirect communication | 35(3.9%) | | 19(5.3%) | 16(2.9%) | .01 |
| Direct communication | 272(30.2%) | | 91(25.5%) | 181(33.3%) |  |
| **Intent to die** |  | |  |  |  |
| No intent/minimal | 125(13.9%) | | 70(19.6%) | 55(10.1%) |  |
| Definite intent/ambivalence | 220(24.4%) | | 92(25.8%) | 128(23.5%) | <.001 |
| Serious/extreme intent | 556(61.7%) | | 195(54.6%) | 361(66.4%) |  |
| **Method of self-harm** |  | |  |  |  |
| Pesticide | 403(44.7%) | | 195(54.6%) | 208(38.2%) |  |
| Ingestion of toxic chemicals | 291(32.3%) | | 76(21.3%) | 215(39.5%) | <.001 |
| Ingestion of medication | 155(17.2%) | | 57(16.0%) | 98(18.0%) |  |
| Others (gunshot and jumping from heights) | 46(5.1%) | | 25(7.0%) | 21(3.9%) |  |
| Ingestion of medication plus pesticides | 6(0.7%) | | 4(1.1%) | 2(0.4%) |  |

*Note*: BDI = Beck Depression Inventory; BHS = Beck Hopeless Scale; BSI = Beck Scale for Suicide Ideation; £1=206 Pak Rupees in Jul 2019

**Table 7**

| **Comparison of severity levels of scores between genders** | | | | |
| --- | --- | --- | --- | --- |
| **Beck Depression Inventory** | **Total** | **Male** | **Female** | **P-value** |
| Mean ± SD | 25.4 ± 12.2 | 22.8 ± 12.4 | 27.0 ± 11.7 | <.001 |
| Minimal ≤ 13 | 162(18.0%) | 89(24.9%) | 73(13.4%) |  |
| Mild 14 to 19 | 143(15.9%) | 62(17.4%) | 81(14.9%) | <.001 |
| Moderate 20 to 28 | 263(29.2%) | 103(28.9%) | 160(29.4%) |  |
| Severe ≥ 28 | 333(37.0%) | 103(28.9%) | 230(42.3%) |  |
| **Beck Hopelessness Inventory** |  |  |  |  |
| Mean ± SD | 9.5 ± 6.0 | 8.1 ± 5.8 | 10.4 ± 6.0 | <0.001 |
| Minimal <=3 | 262(29.1%) | 109(30.5%) | 153(28.1%)- |  |
| Mild 4 to 8 | 181(20.1%) | 99(27.7%) | 82(15.1%) | <.001 |
| Mod 9 to 14 | 191(21.2%) | 77(21.6%) | 114(21.0%) |  |
| Severe >15 | 267(29.6%) | 72(20.2%) | 195(35.9%) |  |
| **Beck Suicidal Ideation Scale** |  |  |  |  |
| Median [IQR] | 9 [2, 17] | 7 [0, 14] | 11 [3, 19] | <.001 |
| Low | 379(42.1%) | 177(49.6%) | 202(37.1%) | <.001 |
| High | 522(57.9%) | 180(50.4%) | 342(62.9%) |  |
